# Supplementary material for: Metabolt: An In-Situ Instrument to Characterize the Metabolic Activity of Microbial Soil Ecosystems Using Electrochemical and Gaseous Signatures
Source: Sensors (Basel). 2020 Aug 11;20(16):4479. doi: 10.3390/s20164479 (PMC7472036; doi:10.3390/s20164479)
Supplement: Supplementary file 1 [file sensors-20-04479-s001.pdf]

sensors

MDPI

1. Mechanical Design

Table S1. Physical dimensions of the Metabolt.

Component	External Dimensions (mm)
Experiment container	94.2 × 64.2 × 64
Experiment container lid	90 × 60 × 37
Experiment container casing	134.8 × 98.4 × 64
Experiment containers w/ lid and casing	134.8 × 133.4 × 107
Electronic box	200 × 110 × 57

The components of the Metabolt: Incubator with two experiment containers and the electronic box were designed with the Dassault Systemes® SOLIDWORKS® 3D CAD design software and 3D-printed with the Ultimaker Extended 2+. The printed parts were further treated with the Smooth-On XTC-3D® high-performance 3D-print coating to fill any leak pores and finished with the Montana GOLD spray paint.

The experiment container design comprehensively accommodates all the sensors and the probes to make it compact and easy to replace in the event of a malfunction or a defect due to corrosion. The two copper sheet electrodes were positioned against the long end of the container walls, forming a geometrical cell constant of K = 0.6, sensitive enough to accurately resolve the soil electrical conductivity, which is generally in the lower conductivity range of few tens to hundreds of μS/cm.

The DS18B20 temperature sensors, the redox and the reference probes, the oxygen and the carbon dioxide sensors were mounted on a lid that encloses the container such that the sensors and the probes dig into the experiment sample while the sensing area of the gas sensors stays 10 mm away from the top surface of the sample, allowing the gas medium in between to be analyzed for the respiratory, metabolic footprint concentrations.

The electronic box has external interfaces with the experiment containers for sending and receiving the signals and a USB communication with a PC for the system software update and the data transmission. It also allows the user to select the power mode of the operation of the instrument with a slider switch; provide the accessibility to a micro-SD memory storage device, and replace the in-built batteries.

2. Electrical Design and System Software

The core of the electronic box is the Whitebox Labs Tentacle Shield for the Arduino and an Arduino Mega 2560 Rev 3. The Tentacle Shield houses the three Atlas Scientific EZO™ circuits: an EC, and the two ORP circuits that are galvanically isolated from each other.

These circuits were programmed to communicate with the ATmega328 processor sequentially via a universal I²C bus. However, from the two experiment containers, we have two EC signals and six ORP signals. To avoid stacking multiple tentacle shields to accommodate more Atlas Scientific EZO™ circuits that would eventually increase the weight, power and cost, Metabolt uses the sequential signal routing approach with the 2:1 and 4:1 bidirectional multiplexer/demultiplexers. The routing part of the circuit was built on a prototyping breadboard and mounted in the casing of the experiment containers, such that only the signal from one of the sensors/probes is transmitted at a time. Redox potential signals from each of the experiment containers were routed to a different ORP circuit to avoid any possible ground looping while the EC circuit was shared. The data lines of the two DS18B20 temperature sensors, one from each experiment, were connected to a single digital pin of the Arduino and programmed to communicate via a universal I²C bus. The oxygen and the carbon dioxide sensors communicate directly with the Arduino by the TX/RX TTL serial communication via the three external TX/RX pins and the two software serial ports.

In addition, the electronic box houses a 4 x 1.5 V AA battery pack, serially connected as a 6 V equivalent power supply, that runs through a slider switch for the user power mode selection and

then to the V_{in} pin of the Arduino, where it is regulated to a 5 V. A micro-SD shield was integrated, providing the user with access to the unprocessed raw data of the experiments and the three status LEDs to monitor the progress. The experiment containers and the electronic box share the electrical connections through a male–female DB-25 cable that plugs into the female DB-25 ports installed on the experiment containers casing and the electronic box.

The ATmega328 processor of the Arduino Mega 2560 Rev 3 controls the entire commanding, processing and the data management protocols. The source code of the system software was developed in the Arduino IDE and can be updated real-time by connecting the Arduino USB port to a PC. The system software was designed systematically to be executed in four stages. 1) Sensor setup, 2) Calibration, 3) Data acquisition and transmission, and 4) Hibernation. Sensor setup prepares the sensors by sending the proper commands regarding the sensor status, output format, etc. Calibration stage assigns the calibration values to the sensors and circuits, if any. It also test-runs the sensors for a few seconds to make sure that everything is in order and ready to conduct the experiments. Data acquisition and transmission is handled by sending the data request commands to the sensors and circuits and after a short processing time delay, the individual variables receive the data in a specified format. Once all the eight measurements were carried out, the data were formatted as the comma-separated values and transmitted to the PC and/or stored in the micro-SD card. After a few allowed iterations at a specified frequency of the data acquisition and transmission, the instrument was hibernated for a specified time to save power and automatically woke up to continue the process. The timing, delays and the counters were managed by the in-built Arduino library function, *millis()*.

3. Data Management Modes

Metabolt supports the two modes by which the data from the experiment are utilized. Depending on the user preferences, nature of the operation, and the mobility and power constraints, the experiment data can either be transmitted to a PC through the USB data connection and/or can be stored locally in the in-built memory (*.csv/.txt/etc.)*.

The local storage mode is simple and straight-forward but allows accessing the data after the completion of the experiments or during the hibernation time to avoid any data loss. On the other hand, the PC mode allows visualizing the data real-time with any serial data acquisition tool and saving in any suitable format (*.csv/.txt/etc.)*. A copy of the unprocessed data is also maintained in the micro-SD storage in this mode.

The data storage requirements for the Metabolt are small: less than 500 kB for a 10- day experiment.

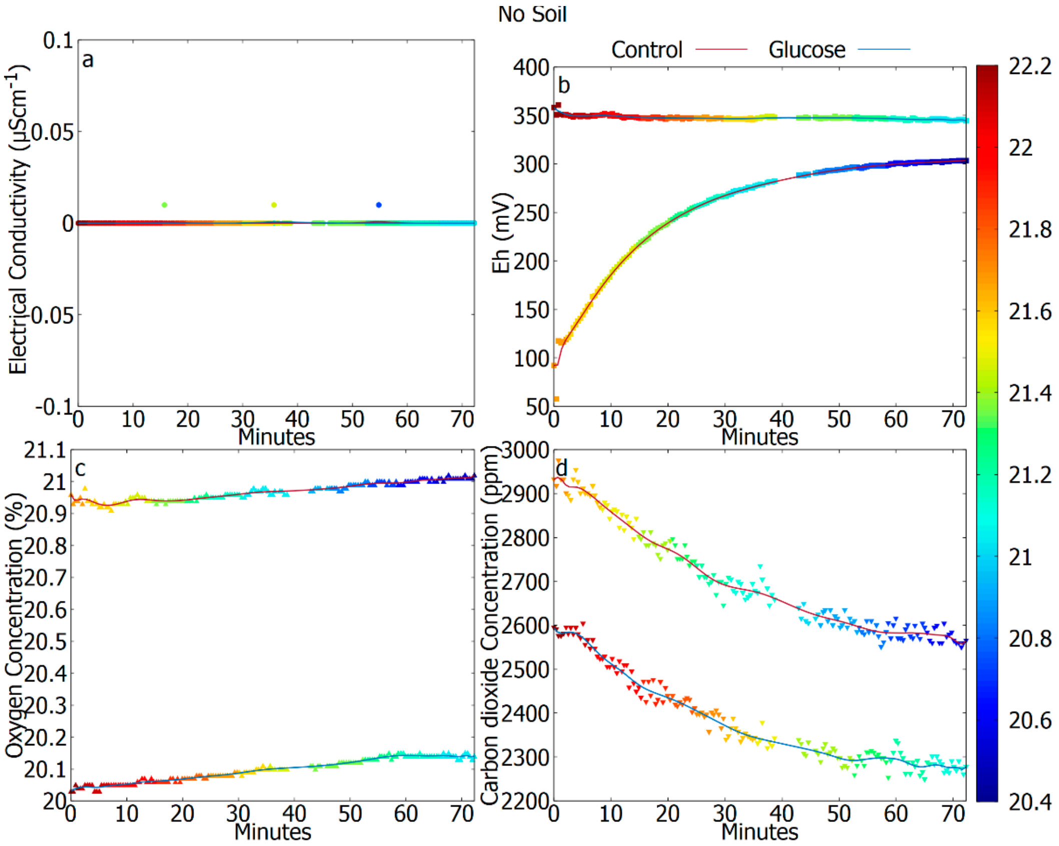

Figure S1. Results of (**a**) Electrical Conductivity (EC), (**b**) Redox potential (Eh), (**c**) Oxygen concentration, (**d**) Carbon dioxide concentration measurements with no soil in experiment containers for 70 min during the afternoon between 15:47 and 16:57. Electrical conductivity shows no variation due to absence in contact between electrodes, redox potential just showing arbitrary values with exposure to air and the oxygen and carbon dioxide concentrations reaching equilibrium with the ambient environment and a gradual lowering in temperature. The color bar represents the air temperature (°C).

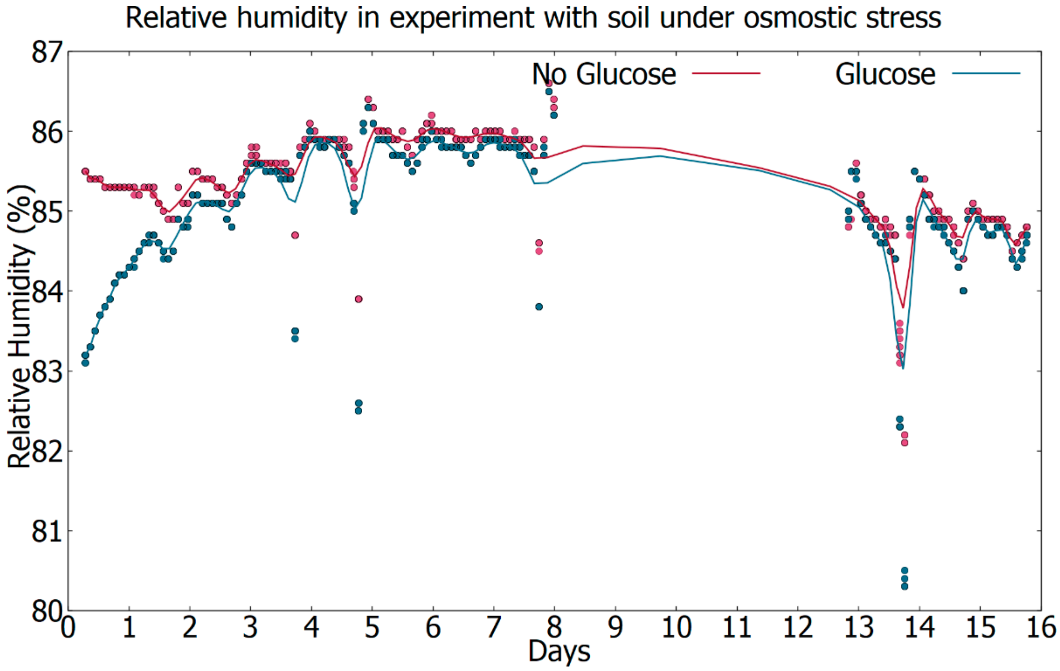

Figure S2. Relative humidity in the headspace with the application of 25% $CaCl_2$ brine (osmotic stress) showing the water activity equivalence for control (red) and glucose (blue) experiments with the soil under osmotic pressure.

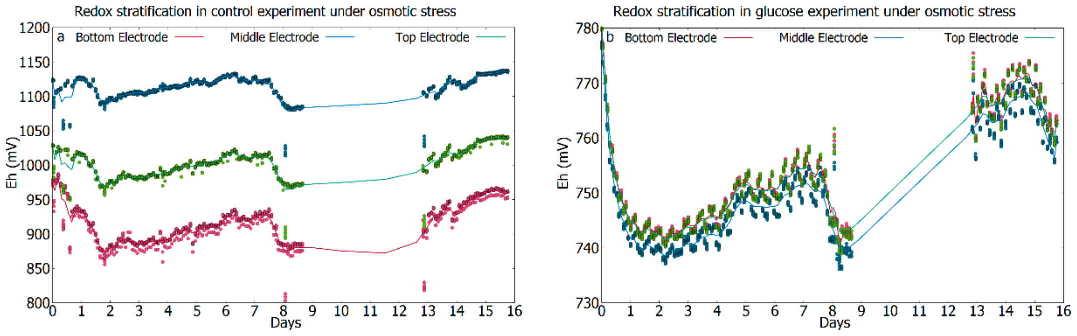

(**a**) (**b**)

Figure S3. Redox potential measurements in the incubated soil under osmotic stress, showing the oxidation-reduction state of the experimental samples at the different depths (10, 20 and 30 mm from the bottom surface). Stratification in the vertical spatial scale: (**a**) Control with a significant stratification, (**b**) Glucose experiment with a narrow margin.

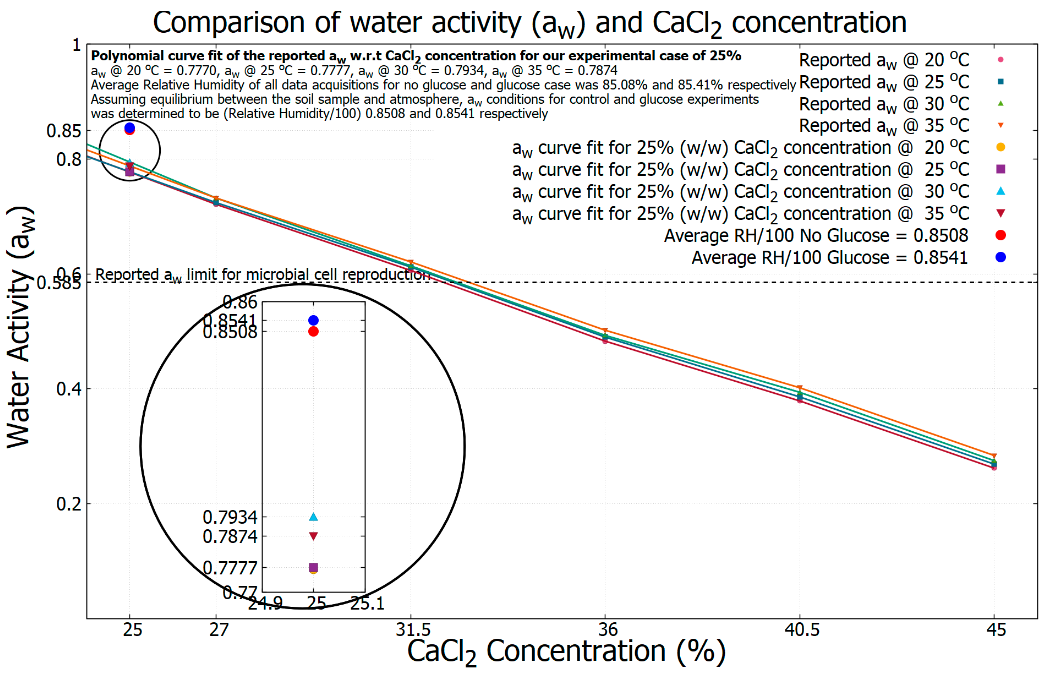

Figure S4. Comparison of the water activity (a_w) and the concentration of $CaCl_2$. Also highlighted (circles) is the predicted water activity for our experimental condition with the 25% concentration of $CaCl_2$ and its correlation with the achieved relative humidity.

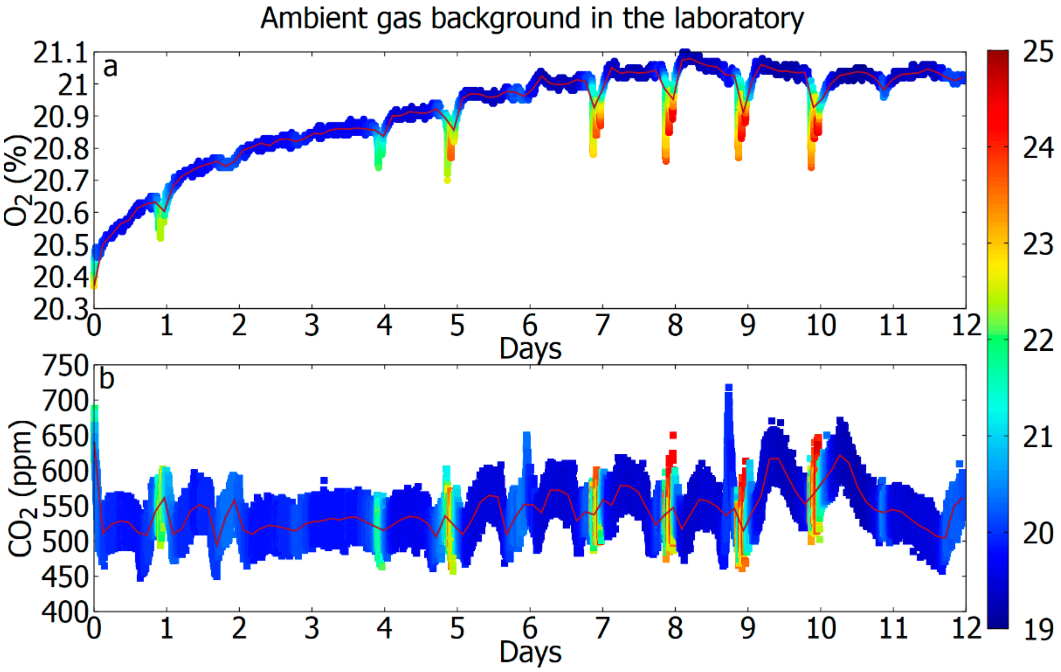

Figure S5. External ambient gas changes in the laboratory during the experiment with the soil under osmotic stress, (**a**) Oxygen concentration and (**b**) Carbon dioxide concentration. The colour bar represents the ambient temperature (°C).

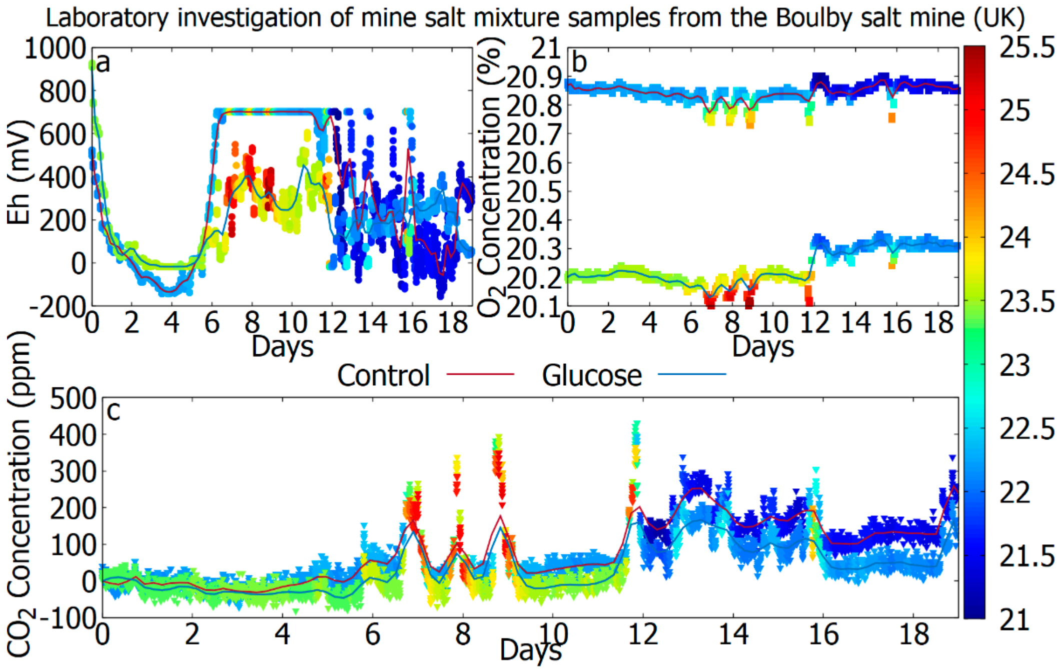

Figure S6. (a) Redox potential, (b) Oxygen concentration, and (c) Carbon dioxide concentration, of a long-time incubation investigation at the laboratory with the mixture of halite, potash and polyhalite salt samples from the Boulby salt mine, UK. The experiment shows a delayed response after 4 days of incubation when the metabolic process in the mine sample is activated enough to be measurable. After activation, the diurnal variation as a function of daily temperature changes could be observed.
